# Detection of carbapenemases *bla*_OXA48_-*bla*_KPC_-*bla*_NDM_-*bla*_VIM_ and extended-spectrum-β-lactamase *bla*_OXA1_-*bla*_SHV_-*bla*_TEM_ genes in Gram-negative bacterial isolates from ICU burns patients

**DOI:** 10.1186/s12941-022-00510-w

**Published:** 2022-05-19

**Authors:** Muhammad Hayat Haider, Timothy D. McHugh, Kerry Roulston, Liã Bárbara Arruda, Zahra Sadouki, Saba Riaz

**Affiliations:** 1 Institute of Microbiology and Molecular Genetics, University of the Punjab, Lahore, Pakistan; 2 Centre for Clinical Microbiology, Division of Infection & Immunity, Royal Free Campus, University College London, London, United Kingdom; 3 525-A Citilab and Research Centre CRC, Faisal Town, Lahore, Pakistan

**Keywords:** Burns, Carbapenemases, *Enterobacteriaceae*, ESBLs, *bla*_OXA48_, *Pseudomonas aeruginosa*, *bla*_VIM_

## Abstract

**Background and objectives:**

Burn patients are highly susceptible to invasion by multidrug-resistant Gram-negative bacteria (MDR-GNB) through post-burn damage. The prevalence of MDR-GNB isolated from burns patients has increased dramatically in the last decade, representing a serious risk to patients admitted to burns units worldwide. The challenges of managing infected burns patients are exacerbated in poor resource settings. This study was designed to develop a pathway for the rapid diagnosis of multidrug-resistant (MDR) Gram-negative infections and identify the bacterial genes including *bla*_OXA1_, *bla*_TEM_, and *bla*_SHV_ encoding ESBLs and *bla*_OXA48_, *bla*_KPC_, *bla*_NDM_, and *bla*_VIM_ encoding carbapenemases from the patient of post burns infection.

**Methods:**

Clinical isolates were collected (August 2017 to August 2018) from Intensive care unit (ICU) of Burn Centre. Antibiotic susceptibility testing and phenotypic detection of ESBLs and carbapenemases was performed by disk diffusion, double disk synergy test (DDST), combination disk test (CDT), and Imipenem + EDTA combined disk test (IMP + EDTA CDT). Polymerase chain reaction (PCR) detection was performed for ESBLs *bla*_OXA1_-*bla*_SHV_-*bla*_TEM_ and carbapenemases genes *bla*_OXA48_-*bla*_KPC_-*bla*_NDM_-*bla*_VIM_

**Results:**

In total, of 170 Gram-negative isolates, 104 (61.2%) were confirmed as multidrug-resistant (MDR); *Pseudomonas aeruginosa* was found to be the most prevalent 43/104 (41.4%), followed by *Klebsiella pneumoniae* 17/104 (16.4%), *Acinetobacter baumannii*12/104 (11.5%), and 6/104 *Proteus mirabilis* (5.8%). All isolates (100%) were resistant to cefotaxime and ceftazidime, while the meropenem resistance was 58.7%. ESBL and carbapenemase genotypes were found to be associated with higher MAR index (0.65–0.88) and MIC (> 32 µg/ml) values *P. aeruginosa* was the major ESBL and carbapenemase producer as determined by phenotypic testing and PCR. *bla*_TEM_ positive isolates among ESBLs producers were predominant 81.8% (27/33), followed by 27.3% *bla*_OXA1_ and *bla*_SHV_, respectively. *bla*_VIM_ positive isolates among carbapenemase producers were predominant 47.7% (21/44), followed by 27.3% *bla*_KPC_, 20.5% *bla*_OXA48_, and 11.4% *bla*_NDM_ positive isolates.

**Conclusions:**

The predominant organism causing burn infections was ESBL and carbapenemase-producing *Pseudomonas aeruginosa*. There are only limited effective antibiotics against such strains. *bla*_VIM_ and *bla*_TEM_ individually and in co-existence with *bla*_KPC_, *bla*_OXA48_, *bla*_SHV_, and *bla*_OXA1_ confer antimicrobial resistance in burns patients. Rapid detection of ESBL and carbapenemase genes will inform treatment strategies improving the outcome for post-burn patients in ICU.

## Background

1]. Many produce extended-spectrum β-lactamases (ESBLs) which confer resistance against third-generation cephalosporins [2], and carbapenemases destroy nearly all β-lactam drugs. Therefore, for successful management, it is necessary to differentiate between ESBL and carbapenemase, producing isolates [3]. Accurate and rapid detection of antimicrobial resistance genes are important in managing the appropriate use of antibiotic not only improving outcomes for individual patients but contributing to antibiotic stewardship minimizing hospitalization costs, morbidity and mortality of severe burns patients [4]. In practice patients at AIMC, Lahore are managed following Hospital Infection Control guidelines and antibiotic stewardship overseen by National Action Plan on Antimicrobial Resistance (National Action Plan on Antimicrobial Resistance (AMR) Pakistan (2017).Phenotypic tests are applied to observe the enzymatic activity of ESBL and carbapenemase, but molecular detection by the polymerase chain reaction (PCR) is the current gold-standard method [5]. Conventional detection techniques are time-consuming and do not fully describe the drug resistance pattern [6]. Multiplex PCR is cost-effective and ensures the detection of several genes in a single reaction and the co-existing genes in a single isolate [7]. Accurate and quick diagnosis of resistance genes can support therapeutic options [8].

*Pseudomonas aeruginosa* is a common infection in burns patients, as are *Acinetobacter baumannii*, *Escherichia coli*, *Klebsiella pneumoniae*, and *Proteus mirabilis* [9, 10]. The global distribution of β-lactams varies with sub-type: SHV type ESBLs primarily detected in *Klebsiella pneumoniae* are distributed in Australia, China, Central and South America. In contrast, the TEM type ESBLs persist in France and North America, and Africa [11, 12]. OXA-type ESBLs conferring resistance in *Pseudomonas aeruginosa* against oxacillin and cephalosporins have been reported from India and Iran. However, very little is known about their worldwide distribution [13, 14]. Genetic variants of all clinically important carbapenemase encoding genes, including *bla*_KPC_, *bla*_NDM_, *bla*_VIM_, and *bla*_OXA48_ can be detected in MDR-GNB [15]. KPC and VIM type carbapenemases have been seen mainly in *K. pneumoniae*, and *P. aeruginosa* strains from the United States [16]. NDM carbapenemases have been reported primarily from India and the United Kingdom, particularly in patients infected with *Enterobacteriaceae* and *Acinetobacter baumannii* strains [17, 18]. OXA-48 carbapenemases are widespread in other European populations. However, Turkey is found with the highest frequency [16, 19]. This study aimed to determine the frequency of ESBL and carbapenemase producing Gram-negative isolates by phenotypic and molecular tests from ICU of burns patients and to use this information to design a diagnostic framework for clinical laboratory management and strengthen the antibiotic stewardship for burn patients.

## Methods

### Study design and data collection

A cross-sectional study was conducted at Jinnah Burns and Reconstructive Surgery Centre (JB&RSC)/Allama Iqbal Medical College (AIMC), Lahore, Pakistan and the Department of Microbiology and Molecular Genetics, University of the Punjab, Pakistan, in collaboration with UCL Centre for Clinical Microbiology, London, United Kingdom. The burns unit consists of 75 beds, and the clinical specimens were collected between August 2017 and August 2018 from 170 patients being treated in the intensive care unit (ICU). The AIMC Ethics Committee approved the study after the submission of the preliminary proposal (ERB-AIMC 40:12 2017). Patients suffering from previous infections receiving any type of antibiotic therapy were excluded. Specimens including wound swabs, blood, sputum, tracheal aspirates, and urine were collected according to AIMS Standard Operating Procedures (AIMS), 2017, from the patients under treatment in the ICU of Burns Center. Specimen enrichment was performed, and subcultures were carried out on Blood, Chocolate, and MacConkey’s agar plates (Oxoid UK). The identification of bacterial isolates was performed using API-20E and 20NE (Biomerieux France) Cephalosporin and carbapenem-resistant Gram-negative isolates were further processed for phenotypic tests and genetic profiling of ESBLs by *bla*_OXA1_-*bla*_SHV_-*bla*_TEM_ and carbapenemases by *bla*_OXA48_-*bla*_KPC_-*bla*_NDM_-*bla*_VIM_ multiplex PCR [7, 20].

## Antimicrobial susceptibility testing

Antimicrobial resistance and susceptibility patterns were analyzed by performing Kirby Bauer’s disk diffusion method, and evaluation of MDR (MDR was defined as acquired non-susceptibility to at least one agent in three or more antimicrobial categories) according to Clinical Laboratory Standards Institute (CLSI, 2017) break points. Antimicrobial discs (Bioanalyse®, Ankara, Turkey) including piperacillin (PIP 100 µg), amoxicillin-clavulanate (AMC 30 µg), piperacillin-tazobactam (TZP 100/10µg), cefepime (FEP 30 µg), ceftazidime (CAZ 30 µg), cefotaxime (CTX 30 µg), doripenem (DOR 10 µg), imipenem (IMP 10 µg), meropenem (MEM 10 µg), amikacin (AK 30 µg), gentamicin (CN 10 µg), tobramycin (TOB 10 µg), ciprofloxacin (CIP 5 µg), levofloxacin (LEV 5 µg), aztreonam (ATM 30 µg), tetracycline and (TE 30 µg) were used for AST profiling of Gram-negative bacterial isolates. Minimum inhibitory concentrations (MICs) were determined for ceftazidime, cefotaxime, imipenem, meropenem. MDR was defined as acquired non-susceptibility to at least one agent in three or more antimicrobial categories.

## Phenotypic detection of ESBLs and carbapenemases

Phenotypic tests, including double-disc synergism test (DDST) and confirmatory combination disc test (CDT) were performed for ESBLs. Double disk synergism for amoxicillin-clavulanate was tested with cefotaxime, ceftazidime, cephradine, aztreonam, cefuroxime, and ceftriaxone. Cefotaxime and ceftazidime disks with and without clavulanic acid were applied for the confirmation of ESBLs. Carbapenemase detection involved IMP + EDTA combined disk test using the combination of imipenem with ethylene-diamide-tetra acetic acid (EDTA) compared to the only imipenem [21].

**Molecular detection of ESBLs** ***bla***_**OXA1**_**-*bla***_**SHV**_**-*bla***_**TEM**_ **and carbapenemases*bla***_**OXA48**_**-*bla***_**KPC**_**-*bla***_**NDM**_**-*bla***_**VIM**_.

DNA extraction was performed by the boiling lysis of a cell suspension of pure bacterial colonies. Previously designed conserved region specific primers OXA-1(F-5′-ATATCTCTACTGTTGCATCTCC-3′, R-5′-AAACCCTTCAAACCATCC-3′), SHV(F-5′-AGGATTGACTGCCTTTTTG-3′, R-5′ATTTGCTGATTTCGCTCG-3′) and TEM(F-5′-ATCAGCAATAAACCAGC-3′, R-5′-CCCCGAAGAACGTTTTC-3′) for ESBLs and OXA-48(F-5′-TTCCCAATAGCTTGATCGC-3′, R-5′-CCATCCCACTTAAAGACTTGG-3′) [20], KPC(F-5′CTGTATCGCCGTCTAGTTCTG-3′, R-5′-AGTTTAGCGAATGGTTCCG-3′), NDM(F-5′-GCATTAGCCGCTGCATT-3′, R-5′-GATCGCCAAACCGTTGG-3′), and VIM(F-5′-TGGCAACGTACGCATCACC-3′, R-5′-CGCAGCACCGGGATAGAA-3′) [3] for carbapenemase detection were used in two separate multiplex PCRs. Thermo scientific master mix ingredients for *bla*_OXA1_-*bla*_SHV_-*bla*_TEM_PCR included buffer 2.5 µl, MgCl_2_ 1.8 µl, dNTPs 0.6 µl, each primer pair 1.2 µl, Taq-polymerase 0.3 µl for a final volume of 25 µl of reaction. PCR 40 cycles with denaturation at 95 °C for 60 s, annealing at 56 °C for 90 s, and extension at 72 °C 60 s. Qiagen PCR ingredients for *bla*_OXA48_-*bla*_KPC_-*bla*_NDM_-*bla*_VIM_PCR included master-mix 10.5 µl, each primer pair 1.5 µl for the final volume of 25 µl of reaction. PCR 40 cycles with denaturation at 95 °C for 30 s, annealing at 50 °C for 30 s, and extension at 72 °C 60 s [7, 20]. PCR accuracy was checked at NCBI (https://www.ncbi.nlm.nih.gov/). PCR amplicons from both assays were visualized by agarose gel electrophoresis with 1% agarose gel and 1X Tris-borate-EDTA (TBE) buffer.

## Results

### Clinical characteristics

In total, 170 clinical isolates were collected, out of which sixty-six were excluded (duplicate, non-MDR etc.), and n = 104 were MDR Gram-negative bacterial pathogens of post-burn infections in ICU patients. *Pseudomonas aeruginosa* 43/104(41.4%) was found to be the most prevalent infectious isolate, followed by *Klebsiella pneumoniae* 17/104 (16.4%), *Acinetobacter baumannii*12/104(11.5%), and 6/104 *Proteus mirabilis* (5.8%). Antimicrobial sensitivity testing confirmed 100% resistance against aztreonam, cefotaxime, ceftazidime, and amikacin. Piperacillin and cefepime resistance were observed in 96.2% and 92.3% isolates, respectively. Resistance against carbapenems was lower; meropenem (58.7%), imipenem (57.7%), and doripenem (56.7%). Colistin resistance was observed in 5.8% isolates (Table 1). The multiple antibiotic resistance (MAR) index values ranged between 0.65 and 0.88. ESBL and carbapenemase genotypes were found to be associated with a higher MAR index (0.65–0.88) and MIC (> 32 µg/ml) values (Tables 2 and 3).

**Table 1:** Antimicrobial resistance patterns of Gram-negative bacterial pathogenic strains isolated from burn patients

Antibiotics	Antimicrobial resistant isolates n = 104 (%)					
	***Pseudomonas***	***Klebsiella***	***Acinetobacter***	***Proteus***	**Others**	**Total**
β-lactamas						
Aztreonam (ATM)	50 (48.1)	19 (18.3)	13 (12.5)	10 (9.6)	12 (11.5)	104 (100)
Piperacillin (PIP)	48 (46.2)	18 (17.3)	13 (12.5)	10 (9.6)	11 (10.6)	100 (96.2)
Piperacillin-tazobactam (TZP)	40 (38.5)	14 (13.5)	10 (9.6)	9 (8.7)	7 (6.7)	80 (76.9)
Amoxycillin-clavulanate (AMC)	46 (44.2)	14 (13.5)	12 (11.5)	7 (6.7)	10 (9.6)	89 (85.6)
Cephalosporins						
Cefotaxime (CTX)	–	19 (18.3)	–	10 (9.6)	12 (11.5)	104 (100)
Ceftazidime (CAZ)	50 (48.1)	19 (18.3)	13 (12.5)	10 (9.6)	12 (11.5)	104 (100)
Cefepime (FEP)	45 (43.3)	18 (17.3)	12 (11.5)	9 (8.7)	12 (11.5)	96 (92.3)
Carbapenems						
Doripenem (DOR)	31 (29.8)	11 (10.6)	5 (4.8)	4 (3.8)	8 (7.7)	59 (56.7)
Imipenem (IMI)	28 (26.9)	12 (11.5)	4 (3.8)	5 (4.8)	11 (10.6)	60 (57.7)
Meropenem (MEM)	32 (30.7)	11 (10.6)	5 (4.8)	4 (3.8)	9 (8.7)	61 (58.7)
Aminoglycosides						
Amikacin (AMK)	50 (48.1)	19 (18.3)	13 (12.5)	10 (9.6)	12 (11.5)	104 (100)
Gentamicin (GEN)	41 (39.4)	17 (16.4)	13 (12.5)	9 (8.7)	11 (10.6)	91 (87.5)
Tobramycin (TOB)	40 (38.5)	18 (17.3)	12 (11.5)	8 (7.7)	12 (11.5)	90 (86.5)
Quinolones						
Ciprofloxacin (CIP)	40 (38.5)	18 (17.3)	11 (10.6)	10 (9.6)	8 (7.7)	87 (83.7)
Levofloxacin (LEV)	42 (40.4)	16 (15.4)	13 (12.5)	9 (8.7)	9 (8.7)	89 (85.6)

Only mentioned resistance %

**Table 2:** Frequency distribution of ESBLs genotypes and their association with antimicrobial resistance among PCR positive isolates from burn patients

ESBLs genotypes	Isolates	N = 33	CTX (MIC µg/ml)	CAZ (MIC µg/ml)	MAR Index
OXA type	*Pseudomonas aeruginosa*	2	> 16	> 32	0.82
	*Pseudomonas fluorescence*	1	> 32	> 32	0.71
	*Enterobacter cloacae*	1	> 16	> 32	0.88
	*Escherichia coli*	1	> 16	> 32	0.82
TEM type	*Pseudomonas aeruginosa*	7	16 to > 32	> 32	0.71 to 0.88
	*Acinetobacter baumannii*	4	16 to > 32	32 to > 64	0.71 to 0.88
	*Klebsiella pneumoniae*	2	16 to > 32	32 to > 64	0.76
	*Escherichia coli*	1	> 16	> 32	0.82
	*Enterobacter cloacae*	1	> 16	> 32	0.82
	*Proteus mirabilis*	1	> 16	> 32	0.82
SHV type	*Escherichia coli*	1	> 32	> 32	0.88
OXA-TEM type	*Acinetobacter baumannii*	1	> 128	> 32	0.71
	*Klebsiella pneumoniae*	1	> 8	> 16	0.76
	*Serratia liquefaciens*	1	> 32	> 16	0.65
TEM-SHV type	*Klebsiella pneumoniae*	3	16 to > 32	> 32	0.76 to 0.82
	*Acinetobacter baumannii*	2	16 to > 64	16 to > 64	0.76 to 0.82
	*Pseudomonas aeruginosa*	1	> 32	> 32	0.82
	*Proteus vulgaris*	1	> 16	> 32	0.71
OXA-TEM-SHV type	*Pseudomonas aeruginosa*	1	> 64	> 128	0.82

**Table 3:** Frequency distribution of carbapenemase genotypes and their association with antimicrobial resistance among PCR positive isolates from burn patients

Carbapenemase genotypes	Isolates	N = 44	IMP (MIC µg/ml)	MEM (MIC µg/ml)	MAR Index
OXA-48	*Pseudomonas aeruginosa*	4	> 8	4 to > 8	0.82 to 0.88
	*Klebsiella pneumoniae*	2	2 to > 8	> 4	0.76
	*Acinetobacter baumannii*	1	> 16	> 8	0.82
	*Pseudomonas fluorescence*	1	> 8	> 8	0.82
KPC type	*Escherichia coli*	4	4 to > 16	2 to > 8	0.82 to 0.88
	*Enterobacter cloacae*	3	4 to > 16	2 to > 8	0.82 to 0.88
	*Klebsiella pneumoniae*	3	4 to > 8	2 to > 4	0.82 to 0.88
NDM type	*Klebsiella pneumoniae*	1	> 4	> 4	0.82
	*Klebsiella oxytoca*	1	> 8	> 8	0.82
	*Acinetobacter baumannii*	1	> 8	> 8	0.76
	*Acinetobacter pittii*	1	> 8	> 8	0.76
	*Proteus mirabilis*	1	1	> 2	0.76
VIM type	*Pseudomonas aeruginosa*	11	4 to > 16	2 to > 16	0.71 to 0.88
	*Citrobacter freundii*	2	2 to > 4	2 to > 4	0.76 to 0.88
	*Proteus mirabilis*	2	8 to > 16	> 4	0.82 to 0.88
	*Klebsiella oxytoca*	1	> 2	> 1	0.82
	*Klebsiella pneumoniae*	1	> 8	> 4	0.76
	*Pseudomonas putida*	1	> 16	> 16	0.88
	*Serratia marcescens*	1	> 4	> 4	0.76
KPC-VIM type	*Proteus mirabilis*	1	> 8	> 4	0.76
OXA48-KPC-VIM type	*Enterobacter cloacae*	1	> 16	> 16	0.82

## ESBLs and carbapenemases

ESBL detection by DDST and CDT revealed 21.2% (22/104), whereas genotyping by multiplex PCR yielded 31.7% (33/104) positive isolates. Carbapenemase detection by IMP-EDTA combination disk test resulted in 52.9% (55/104), and multiplex PCR yielded 42.3% (44/104) positive isolates (Table 4). Phenotypic testing revealed 4.8% (number) isolates, including *P. aeruginosa*, *K. pneumoniae* and *P*. *mirabilis*, positive for both the ESBLs and carbapenemases. *bla*_TEM_ positive isolates were predominant at 81.8% (27/33), followed by 27.3% *bla*_OXA1_ and *bla*_SHV_, respectively. *bla*_SHV_-*bla*_TEM_ co-existence was observed in 21.2%, followed by *bla*_OXA1_- *bla*_TEM_ in 9.1% and *bla*_OXA1_-*bla*_SHV_-*bla*_TEM_ in 3% isolates (Table 2).

**Table 4:** Bacteriological profiling, phenotypic and molecular testing of ESBLs and carbapenemase producing Gram-negative isolates from burn patients

Burns isolates	N (%)	ESBLs n (%)		Carbapenemases n (%)	
		**DDST/CDT**	**PCR**	**IMP + EDTA**	**PCR**
*Pseudomonas aeruginosa*	43 (41.4)	12 (54.6)	11 (33.3)	22 (40)	15 (34.1)
*Klebsiella pneumoniae*	17 (16.4)	3 (13.6)	6 (18.2)	8 (14.5)	7 (15.9)
*Acinetobacter baumannii*	12 (11.5)	4 (18.2)	7 (21.2)	3 (5.5)	2 (4.5)
*Proteus mirabilis*	6 (5.8)	1 (4.5)	1 (3)	5 (9.1)	4 (9.1)
*Pseudomonas putida*	5 (4.8)			2 (3.6)	1 (2.3)
*Enterobacter cloacae*	4 (3.9)		2 (6.1)	3 (5.5)	4 (9.1)
*Escherichia coli*	4 (3.9)		3 (9.1)	4 (7.3)	4 (9.1)
*Proteus vulgaris*	4 (3.9)	1 (4.5)	1 (3)	1 (1.8)	
*Citrobacter freundii*	2 (1.9)			2 (3.6)	2 (4.5)
*Klebsiella oxytoca*	2 (1.9)			2 (3.6)	2 (4.5)
*Pseudomonasfluorescence*	2 (1.9)	1 (4.5)	1 (3)	1 (1.8)	1 (2.3)
*Acinetobacter pittii*	1 (0.9)			1 (1.8)	1 (2.3)
*Serratia liquefaciens*	1 (0.9)		1 (3)		
*Serratia marcescens*	1 (0.9)			1 (1.8)	1 (2.3)
Total	104 (100)	22 (21.2)	33 (31.7)	55 (52.9)	44 (42.3)

1). *bla*_VIM_ positive isolates were predominant 47.7% (21/44) followed by 27.3% *bla*_KPC_, 20.5% *bla*_OXA48_, and 11.4% *bla*_NDM_ positive isolates. *bla*_KPC_-*bla*_VIM_ and *bla*_OXA48_-*bla*_KPC_-*bla*_VIM_ co-existence was observed in *P*. *mirabilis* and *E*. *cloacae*, respectively (Table 3).

**Fig. 1:**
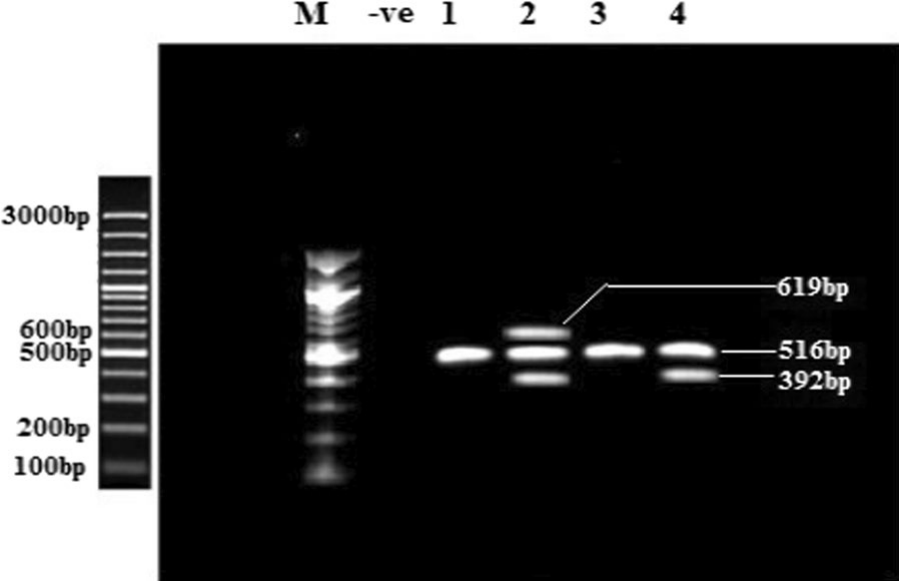
Agarose gel electrophoresis of multiplex PCR for ESBLs genes detection including *bla*_OXA1_, *bla*_TEM_, *bla*_SHV_. M: 100 bp DNA marker (Thermo-scientific), -ve: blank controls, 1: *bla*_OXA1_ (619 bp), 2: multiple genes including *bla*_OXA1_ (619 bp), *bla*_TEM_ (516 bp), and *bla*_SHV_ (392 bp), 3: *bla*_TEM_ (516 bp), 4: multiple genes including *bla*_TEM_ (516 bp), and *bla*_SHV_ (392 bp)

**Fig. 2:**
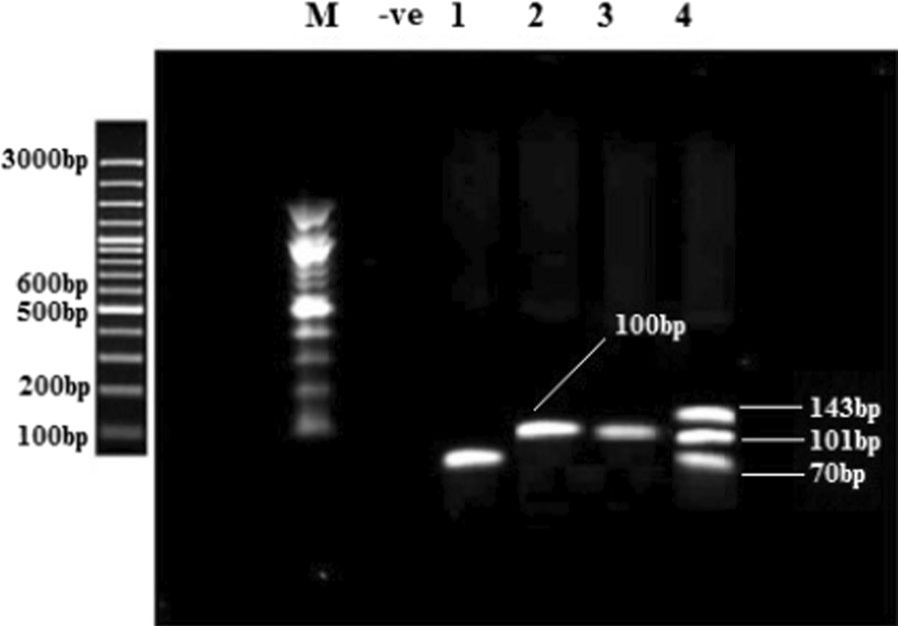
Agarose gel electrophoresis of multiplex PCR for carbapenemases genes detection including *bla*_OXA−48_, *bla*_NDM_, *bla*_KPC_, and *bla*_VIM_. M: 100 bp DNA marker (Thermo-scientific), -ve: blank controls, 1: *bla*_OXA−48_ (70 bp), 2: *bla*_NDM_ (100 bp), 3: *bla*_KPC_(101 bp), 4: Multiple genes including*bla*_VIM_ (143 bp), *bla*_KPC_ (101 bp), and *bla*_OXA−48_ (70 bp)

Carbapenemase PCR amplicons were identified by the expected molecular size of the amplified fragments (Fig. 2). KPC carbapenemases genes were found co-existing with ESBLs in 4.8% isolates, including three *E. coli* strains with *bla*_OXA1_-*bla*_KPC_, *bla*_TEM_-*bla*_KPC_, and *bla*_SHV_ -*bla*_KPC_, and two *E. cloacae* strain with *bla*_OXA1_- *bla*_KPC_ and *bla*_TEM_-*bla*_KPC_ genotypes.

## Discussion

Burn wounds being managed in surgical and intensive care units are at high risk of exposure to multidrug-resistant (MDR) bacterial pathogens [22]. The current study was designed to describe the epidemiology of MDR-Gram negative bacteria in this population and as such did not directly collect clinical outcome or antibiotic usage data. However, we have demonstrated a high level of drug resistance among Gram-negative bacteria isolated from burns patients in the burns ICU Centre of Lahore, Pakistan. Bacteriological profiling of Gram-negative isolates has shown *P. aeruginosa* as the most prevalent isolate followed by *Enterobacteriaceae*, particularly *K. pneumoniae* and *A. baumannii*. Similar findings have been reported from India and Iran previously [2, 4]. These strains appeared with higher MAR values attributed to ESBLs, carbapenemases and other genetic factors [12, 15]. The empirical treatment preceding the diagnosis as an infection control strategy in ICUs is a frequently reported factor behind the inadequate response of third-generation cephalosporins and carbapenems [23]. Burns patients are also given intravenous injections of carbapenems before bacterial culture [24]. The higher proportions of aminoglycoside and quinolone-resistant strains mark the possibility of resistance factors besides ESBLs and carbapenemases [6, 18].

We have observed that isolates from wounds are mostly associated with *P. aeruginosa*, and VIM-like carbapenemases, which agrees with studies reported from Algeria and China [24, 25]. OXA-like carbapenemases are an important cause of the acquisition of carbapenemases in *A. baumannii* isolated in Iran [23]. NDM-producing *Enterobacteriaceae* have been frequently reported from India, Pakistan, and China [17, 26]. Surgical site infections have been studied in Vietnam, where SHV was the most prevalent ESBL followed by TEM, while NDM was the only carbapenemase observed in *E. coli* strains [8]. TEM followed by SHV-like ESBLs are associated with *A. baumannii* isolates from burn patients in Iraq with more than 70% resistance against cephalosporins and carbapenems [27].

There are minimal data on antimicrobial resistance and molecular profiling of ESBLs and carbapenemases from burns patients in Pakistan. The co-existence of more than one genetic variant encoding ESBLs produces a masking effect decreasing the diffusion or permeability of the antibiotic, leading to false-negative results [6, 11]. The false reporting of ESBL positive isolates leads to therapeutic failure [28]. Phenotypic tests and PCRs also confirmed the co-existence of carbapenemases and ESBLs encoding genes. The IMP + EDTA combination disc test revealed a higher frequency of carbapenemase producers than ESBLs. Carbapenemases PCR positive isolates were less than the IMP + EDTA test as the PCR detects only primer specific genetic determinant [5]. Therefore, we propose a fstrategy for the rapid detection of MDR-GNB that both phenotypic and molecular tests should be used simultaneously [29]. The carbapenems resistant, but PCR negative isolates mark other genetic variants of ESBLs and carbapenemases or non-enzymatic resistance factors [30].

## Conclusions

In conclusion, the rates of ESBLs and carbapenemases producing strains among ICU burns patients is high. In our setting *Pseudomonas aeruginosa* is the frequently ESBLs and carbapenemase-producing strain isolated from burns patients. VIM carbapenemases, TEM ESBLs individually and in co-existence with KPC, OXA-48, and SHV and OXA-1 ESBLs confer antimicrobial resistance in burns patients. Here we report the development of a pragmatic diagnostic strategy for ESBLs and carbapenemase-producing clinical isolates which provides a presumptive diagnosis to inform rapidly the selection of antibiotic therapy: an exceptional diagnostic and clinical strategy is required to combat such an alarming situation to improve healthcare and control the spread of infections. Therefore, it is necessary to properly manage phenotypic and molecular methods to provide complete resistance profiles to ensure appropriate antibiotic administration.

## Data Availability

Not applicable.

## Abbreviations

MDR-GNB: Multidrug-resistant gram-negative bacteria
ICU: Intensive care unit
ESBLs: Extended-spectrum beta-lactamase
AST: Antibiotic susceptibility testing
DDST: Disk diffusion, double disk synergy test
CDT: Combination disk test
IMP + EDTA CDT: Imipenem + EDTA combined disk test
PCR: Polymerase chain reaction
MAR: Multiple antibiotic resistance
MIC: Minimum inhibitory concentrations
