# Correction: Detection of carbapenemases* bla*_OXA48_-*bla*_KPC_-*bla*_NDM_-*bla*_VIM_ and extended-spectrum-β-lactamase* bla*_OXA1_-*bla*_SHV_-*bla*_TEM_ genes in Gram-negative bacterial isolates from ICU burns patients

**DOI:** 10.1186/s12941-022-00519-1

**Published:** 2022-07-07

**Authors:** Muhammad Hayat Haider, Timothy D. McHugh, Kerry Roulston, Liã Bárbara Arruda, Zahra Sadouki, Saba Riaz

**Affiliations:** 1 Institute of Microbiology and Molecular Genetics, University of the Punjab, Lahore, Pakistan; 2 Centre for Clinical Microbiology, Division of Infection & Immunity, Royal Free Campus, University College London, London, UK; 3 525-A Citilab and Research Centre CRC, Faisal Town, Lahore, Pakistan; 4 Wellcome Connecting Science, Wellcome Genome Campus, Hinxton, CB10 1RQ UK

## Correction to: Ann Clin Microbiol Antimicrob (2021) 21: 1–8 10.1186/s12941-022-00510-w

Following publication of the original article [1], the author noticed an error in the affiliation of the co-author, “Liã Bárbara Arruda” in the published version. Liã Bárbara is affiliated in “Wellcome Connecting Science, Wellcome Genome Campus, Hinxton, CB10 1RQ, UK”. This has been corrected with this erratum.

The Competing interests section should also have stated that Timothy D. McHugh is an Editor-in-Chief of Annals of Clinical Microbiology and Antimicrobials and was not involved in the peer review process for the manuscript.
